# Erythema and Burning Pain in the Vulva: A Possible Phenotype of Erythromelalgia

**DOI:** 10.1155/2011/374167

**Published:** 2011-04-13

**Authors:** Elisabeth Johnson, Priya Iyer, Alisa Eanes, Denniz Zolnoun

**Affiliations:** ^1^Pelvic Pain Research Unit, Division of Advanced Laparoscopy and Pelvic Pain, Department of Obstetrics and Gynecology, School of Medicine, University of North Carolina, Chapel Hill, NC 27599-7570, USA; ^2^School of Nursing, Georgia State University Atlanta, GA 30302-4019, USA; ^3^Department of Obstetrics and Gynecology, University of North Carolina, Chapel Hill, NC 27599-7570, USA; ^4^Drexel University, College of Medicine, Philadelphia, PA 19102, USA; ^5^Department of Obstetrics and Gynecology, and Center for Neurosensory Disorders, University of North Carolina, Chapel Hill, NC 27599-7570, USA

## Abstract

We report a case of burning vulvar pain accompanied by erythema responding to an oral combination of a benzodiazepine and a beta blocker. The positive response to two medication classes used in the treatment of erythromelalgia supports the possibility of a localized manifestation of this disorder in the genital region.

## 1. Introduction

Erythromelalgia (EM) is a rare disorder characterized by episodic erythema of the skin associated with increase in temperature and burning pain. While EM is thought to primarily affect the extremities, particularly the hands and the feet, it has been described in other parts of the body such as the head, neck, and scrotum.

## 2. Case Presentation

**Figure 1:**
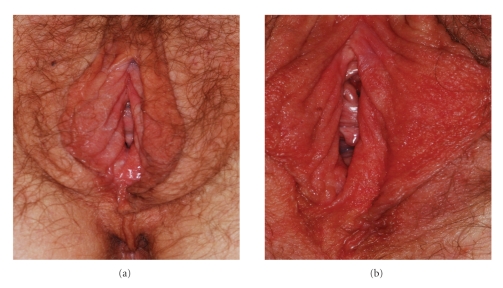
After treatment with beta-blocker (a). Acute flare (b).

A 67-year-old Caucasian female presented for evaluation of persistent burning pain and itching in the vulvar region accompanied by marked erythema and warmth to the touch (Figure 1). Her symptoms first appeared in childhood but increased in frequency and severity after menopause. During her reproductive years, she had experienced a myriad of urologic and gynecological symptoms. In her 20s, the preponderance of her symptoms involved her bladder, and she was subsequently diagnosed with interstitial cystitis. In her 30s, her gynecologic symptoms became more prominent and she was ultimately diagnosed with endometriosis for which she underwent a hysterectomy and bilateral oophorectomy at age 33. Throughout this time, her unexplained episodic vulvar symptoms were present; however, it was not until menopause that her vulvar symptoms became persistent and the frequency and severity of her flares dramatically increased. She found that the application of cold water and minimizing humidity and warmth in the perineal region were the only ameliorative modalities consistently effective in decreasing the frequency and severity of her “flares.” It is interesting to note that her mother and a maternal aunt had similar lifelong symptoms which they had attributed to horse back riding.

On initial evaluation, the patient had normal appearing vulvar skin and vulvar mucosal changes consistent with the diagnosis of menopausal atrophy. On examination, she experienced diffuse burning and pain when the vulvar skin was gently stroked with a cotton swab. For the purpose of the symptomatic management of worsening burning and dyspareunia, we prescribed a trial of topical estrogen (0.05%) and lidocaine (5%) compounded in hydrophilic petrolatum. While this regimen provided some relief, she continued to experience “flares.” Consequently, the patient was asked to return upon recurrence of a “flare” for evaluation and photographic documentation (Figure 1(a)). In the absence of a known diagnosis, we empirically prescribed bedtime clonazepam (0.5 mg) which improved her overall sense of well being and decreased the intensity of the flares.

While her symptoms were manageable on clonazepam for 3 months, a marked improvement occurred only after a serendipitous change in her antihypertensive regimen. Within 2 weeks of her primary care provider adding a beta blocker (nebivolol) to her medical regimen, she noted a drastic improvement in her symptoms of vulvar burning and accompanying erythema (Figure 1(b)). To date, more than six months after the start of beta-blocker, the redness of her vulvar region has subsided, and her quality of life has dramatically improved.

## 3. Discussion

Given the uncommon occurrence and possible failure to recognize mild symptoms, accurate estimates of the incidence and prevalence of EM are difficult to obtain. It is thought that the condition occurs most commonly in women, and while it can present at any age, it is most often seen in the fifth and sixth decades of life [1]. Though primarily thought to affect the extremities, EM has also been described in the ears, face, and most recently the scrotum [1, 2]. 

The etiology of EM is not well understood and consequently EM is often a diagnosis of exclusion. EM is clinically classified into primary and secondary subtypes [3]. Primary EM is thought to be due to a genetic susceptibility on chromosome 2 that results in a mutation of the gene that codes for voltage gated sodium channels [4–6]. This mutation leads to a dysfunction of vasomotor regulation and results in a shunting process. Symptoms of primary EM usually present early in life are symmetric and alleviated by cold. Secondary EM is thought to arise from medication use, hematologic or connective tissue disorders, or neuropathies [7]. For example, EM associated with thrombocythemia has been described in polycythemia vera [8]. In this setting, symptoms are more likely to be unilateral and initially present as itching and prickling that evolves into severe burning pain and warmth to the touch. Our patient's symptoms appear to be a mix of both primary and secondary EM: she has a lifelong history of symmetric symptoms alleviated by cold and an itching as well as prickling pain that has evolved into severe burning pain during her fifth decade of life. It is important to note that while recognized as an important marker of secondary EM, hemoglobin and platelet counts were not checked in this patient. Her blood work has been monitored by her primary care provider as a part of the general management of her hypertension. To date, there has been no report of clotting disorders or other hematologic conditions that might further complicate the clinical picture of this patient. Additionally, given that this patient presented for the treatment of lifelong vulvar pain, other medications, often used in the treatment of EM such as calcium channel blockers and highdose aspirin were not utilized.

## 4. Conclusion

While there has been recent progress in understanding EM, patients and healthcare providers continue to find the treatment and management quite frustrating. There is no single medication, combination of medications or method of treatment that has been found to be universally helpful in patients suffering from EM [1, 9]. Therefore, providers often rely upon trial and error. 

While a universally effective treatment regimen for EM has not been established, beta blockers and centrally acting agents (such as anticonvulsants and benzodiazepine) are among the numerous medications that have illustrated clinical efficacy in the management of the signs and symptoms of EM [9–12]. Our patient has had remarkable improvement of her lifelong symptoms on a combination of a beta blocker and a benzodiazepine. This improvement has persisted for greater than six months (to date). It is noted that nebivolol, in contrast with other beta blockers, is known to have vasodilatory effects. We cannot postulate a specific mechanism that would account for the dramatic response but hypothesize that differential responses to beta blocker sensitivity that have been observed across individuals may play a role. Our patient's presentation and treatment response suggests that EM may similarly occur in the vulvar region as it does in the scrotum. However, the clinical course and genital manifestation of EM may differ from its presentation in extremity leading to additional diagnostic challenges in genitalia.
